# Risk factors and intestinal microbiota: *Clostridioides difficile* infection in patients receiving enteral nutrition at Intensive Care Units

**DOI:** 10.1186/s13054-020-03119-7

**Published:** 2020-07-13

**Authors:** Daosheng Wang, Danfeng Dong, Chen Wang, Yingchao Cui, Cen Jiang, Qi Ni, Tongxuan Su, Guanzheng Wang, Enqiang Mao, Yibing Peng

**Affiliations:** 1 Department of Laboratory Medicine, Ruijin Hospital, Shanghai Jiao Tong University School of Medicine, No. 197 Ruijin ER Road, Shanghai, 200025 China; 2 Faculty of Medical Laboratory Science, Shanghai Jiao Tong University School of Medicine, No. 197 Ruijin ER Road, Shanghai, 200025 China; 3 Department of Emergency, Ruijin Hospital, Shanghai Jiao Tong University School of Medicine, No.197 Ruijin ER Road, Shanghai, 200025 China

**Keywords:** *Clostridioides difficile* infection, Enteral nutrition, Intensive unit care, Intestinal microbiota, Risk factors, *Bacteroides*

## Abstract

**Background:**

*Clostridioides difficile* infection (CDI) is a leading cause of nosocomial diarrhea. Patients receiving enteral nutrition (EN) in the intensive care unit (ICU) are potentially at high risk of CDI. In the present study, we assessed the risk factors and intestinal microbiome of patients to better understand the occurrence and development of CDI.

**Methods:**

Patients were screened for *C. difficile* every week after starting EN, and their clinical records were collected for risk factor identification. Fecal samples were analyzed using 16S rRNA sequencing to evaluate the intestinal microbiota.

**Results:**

Overall incidence of CDI was 10.7% (18/168 patients). History of cerebral infarction was significantly associated with CDI occurrence (OR, 9.759; 95% CI, 2.140–44.498), and treatment with metronidazole was identified to be protective (OR, 0.287; 95% CI, 0.091–0.902). Patients with EN had lower bacterial richness and diversity, accompanied by a remarkable decrease in the abundance of *Bacteroides*, *Prevotella_9*, Ruminococcaceae, and Lachnospiraceae. Of these patients, acquisition of *C. difficile* resulted in a transient increase in microbial diversity, along with consistent alterations in the proportion of some bacterial taxa, especially Ruminococcaceae and Lachnospiraceae. Upon initiation of EN, patients who were positive for *C. difficile* later showed an enhanced load of *Bacteroides*, which was negatively correlated with the abundance of *C. difficile* when CDI developed.

**Conclusion:**

ICU patients receiving EN have a high prevalence of CDI and a fragile intestinal microbial environment. History of cerebral infarction and prior treatment with metronidazole are considered as vital risk and protective factors, respectively. We propose that the emergence of CDI could cause a protective alteration of the intestinal microbiota. Additionally, *Bacteroides* loads seem to be closely related to the occurrence and development of CDI.

## Background

*Clostridioides difficile*, a Gram-positive, spore-forming anerobic bacterium of the colon, can cause a wide range of illnesses from diarrhea to more severe pseudomembranous colitis [1, 2]. In recent years, there has been a dramatic increase in the incidence and severity of CDI, leading to prolonged hospital stays and significant increased economic burdens, which together have spurred worldwide concern [3]. *C. difficile* infection (CDI) is closely related to antibiotic exposure, which disrupts the endogenous intestinal microbiota and promotes proliferation of *C. difficile* [1, 4]. In addition to antibiotic usage, risk factors for CDI include advanced age, underlying disease, admission to the intensive care unit (ICU), proton pump inhibitor (PPI) treatment, and enteral nutrition (EN) [5–8]. EN, also known as tube feeding, is widely used among patients admitted to ICUs. Due to the increased access of *C. difficile* spores through the feeding tube and the usage of prophylactic treatments with antibiotics or PPIs, patients receiving EN are potentially more vulnerable to CDI [9]. Our previous study also found a significant association between EN and development of CDI in ICU patients [10]. However, the incidence and specific risk factors for CDI in patients with EN have not been comprehensively investigated.

Nonetheless, it has been established that the structure of the intestinal microbiota is closely tied to the development of CDI [11]. ICU patients with EN usually receive consistent diets, and most of them are exposed to broad-spectrum antibiotics and PPIs, which could affect the intestinal microbiota [12]. *C. difficile* itself, however, may also cause distinct alteration of the host microbiome. Therefore, intestinal microbiota in patients receiving EN, along with their interaction with *C. difficile*, need to be further explored.

In the current study, we conducted a prospective study on patients admitted to the ICU with EN. Our objective was to evaluate the incidence and risk factors for CDI in these patients, describe the characteristics of their gut microbiota, and ultimately gain a better understanding of the association between the host microbiome and *C. difficile*.

## Methods

### Study design and clinical data collection

We conducted a prospective study on adult patients admitted to the ICU of Ruijin Hospital (Shanghai, China) between July 2018 and December 2019. All patients who had received EN for at least 1 week were included. Fecal specimens were obtained from each patient at the beginning of EN, every week during EN, and at the onset of diarrhea, if applicable. According to European guidelines [13], CDI was determined by meeting the following two criteria: (1) the occurrence of a positive toxigenic *C. difficile* detection test and (2) the presence of diarrhea characterized by at least three episodes of unformed stools within a 24-h time period. Cultures showing growth of *C. difficile* without any clinical symptoms or toxigenic detection were considered CDC cases.

Clinical epidemiological information for all eligible patients was extracted from patient medical records, including demographics, duration of hospitalization, surgical intervention (within the previous 6 months), mortality, comorbidity, and in-hospital medication. Comorbidity was graded using the Charlson comorbidity index (CCI) and divided into 10 major categories based on related systems. Other common underlying diseases in ICUs were analyzed separately. Laboratory indices, including leukocyte counts, serum albumin levels, and serum creatinine and blood glucose levels, were measured and recorded upon admission. Formulas used in EN and their access routes were also recorded. Antibiotics and PPIs were the most commonly used medications. For CDI patients, medication history was recorded from the time of admission up until the onset of CDI. For *C. difficile*-negative (CDN) patients, data were collected from time of admission up through 2 weeks post-EN, which represented the approximate median number of days passing from start of EN to onset of CDI.

To investigate the gut microbiota features, we recruited 12 healthy individuals from four communities in Shanghai who did not present any gastrointestinal disease or usage of antibiotics in the past month to serve as healthy negative controls. All fecal samples were screened for *C. difficile* and stored at − 80 °C for subsequent DNA extraction.

The present study was approved by the Ethics Committee of Ruijin Hospital, Shanghai, China.

### *C. difficile* detection

Stool samples were analyzed for toxin A/B by enzyme-linked fluorescence assay (ELFA) using a VIDAS automatic analyzer (BioMérieux, Marcy-l’Etoile, France). *C. difficile* isolates were cultured on a *Clostridium difficile* agar base (Oxoid, Basingstoke, UK). Typical colonies were identified based on their odor, appearance, and morphology after Gram staining and confirmed using *gluD* gene detection by polymerase chain reaction (PCR). Purified *C. difficile* isolates were characterized by detection of *toxinA* and *toxinB* genes.

### DNA extraction, 16S rRNA gene sequencing, and data processing

Fecal genomic DNA was extracted from each stool specimen using a TIANamp Stool DNA Kit (Tiangen Biotech, Beijing, China). After quality verification, DNA was submitted to Majorbio Bio-Pharm Technology Co. Ltd. (Shanghai, China) for 16S rRNA gene amplification and sequencing. The hypervariable region V3–V4 of the bacterial 16S rRNA gene was amplified with primer pairs 338F (5′-ACTCCTACGGGAGGCAGCAG-3′) and 806R (5′-GGACTACHVGGGTWTCTAAT-3′). Purified amplicons were sequenced on an Illumina MiSeq platform (Illumina, San Diego, CA, USA). Operational taxonomic units (OTUs) with a 97% similarity cutoff were clustered using UPARSE (version 7.1), and chimeric sequences were identified and removed. The taxonomy of each OTU representative sequence was analyzed by RDP Classifier against the 16S rRNA database (Silva SSU132) using a confidence threshold of 0.7. All processes were performed on a platform (www.i-sanger.com) provided by Majorbio Bio-Pharm Technology Co. Ltd.

### Real-time PCR

Quantitative PCR was performed using the TB Green qPCR Kit (Takara, Tokyo, Japan) and LightCycler 480 Real-Time PCR system (Roche, Shanghai, China). Relative abundance of each bacterium was calculated by the ΔCt method and normalized to total bacteria (16S rRNA). The primer sequences are listed in Table S1.

### Statistical analyses

The results are expressed as medians and quartiles for continuous variables and as frequencies and percentages for categorical variables. The Wilcoxon rank-sum test was used to examine differences in data not normally distributed, including duration of hospitalization, leukocyte count, serum creatinine level, and blood glucose level. Student’s *t* tests were used to compare normally distributed continuous variables, including age, CCI score, and serum albumin. All categorical data were compared by employing a *χ*^2^ test or Fisher’s exact test. A conditional multivariate logistic regression analysis was performed to identify risk factors. All variables with a *P* value < 0.1 from the univariate analysis, along with variables that were identified clinically relevant to CDI in ICU from previous studies [10, 14], were included in the initial regression model. Only variables with a *P* value < 0.1 in the initial model were included in the final multivariate regression model. These analyses were performed with SPSS version 24.0.

The alpha diversity (Chao and Shannon indexes) of the microbiome was calculated at the OTU level on the Majorbio BioTech platform and compared among groups using a Student’s *t* test or paired *t* test. Principal coordinates analysis (PCoA) of the Bray–Curtis distance metric was conducted to evaluate the variability in OTUs among groups, and the differences were tested through Adonis analysis. Linear discriminant analysis effect size (LEfSe) was evaluated from phylum to genus, and the linear discriminant analysis (LDA) score was set at > 4.0. The predominant phyla or genera were also compared among groups using the Wilcoxon rank-sum test or Wilcoxon signed-rank test. Correlations between genus or species relative abundance were calculated using Spearman’s analysis. The *t* tests and Spearman’s correlation tests were processed in GraphPad Prism 5, and the remaining tests were processed using the Majorbio BioTech platform.

Differences were considered significant at *P* < 0.05.

## Results

### Patient population and *C. difficile* detection

**Fig. 1:**
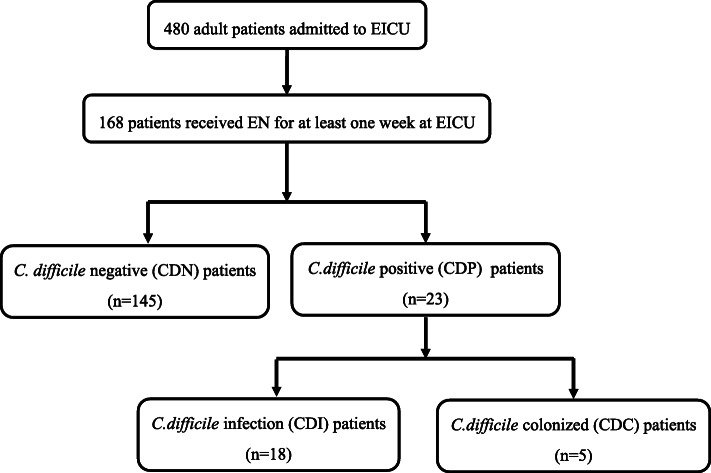
Study flowchart of *Clostridioides difficile* infection (CDI), *C. difficile* colonization (CDC), and *C. difficile*-negative (CDN) patients among the ICU patients. Overall, 168 patients were included in the study and were divided into two groups according to whether they were positive for *C. difficile*. Further grouping was performed according to diarrhea symptoms

A total of 480 adult patients were admitted to the ICU from July 2018 to December 2019 (Fig. 1). Of these patients, 168 had received EN for at least 1 week and were recruited to the present study. The patients were of an average age of 50.5 ± 16.0 (mean ± SD) years, and 31% (52/168) were elderly (> 60 years old). All patients had received antibiotic treatment, and 160 (95.2%) patients had also received PPIs. Nasogastic and nasojejunal placements were two main ways for these patients to receive EN.

We analyzed a total of 695 fecal samples, among which 30 samples from 23 patients revealed positive readings for *C. difficile* (culture or ELFA) (Table S2). Eighteen patients developed diarrhea and were identified to have CDI, while five were defined as *C. difficile* colonization (CDC). Overall, the prevalence of CDI and CDC in ICU patients receiving EN were 10.71% and 2.98%, respectively. The median duration from EN therapy to CDI diagnosis was 12 days (interquartile range, 7–21 days).

### Clinical characteristics and risk factors for CDI in ICU patients with EN

We compared demographics, clinical features, and in-hospital medication between the CDI and CDN groups (Table 1). The results showed that CDI patients were significantly older (median 66 vs. 48 years, *P* = 0.021) and had longer ICU stays (median 30 vs. 20 days, *P* = 0.001). In addition, CDI patients presented distinctly higher CCI scores (median 2.5 vs. 2, *P* = 0.015); however, no significant differences were detected in any comorbidity categories. For other clinically common diseases, we found that a much larger proportion of CDI patients had a history of cerebral infarction (22.2% vs. 3.4%, *P* = 0.006). Laboratory results showed that CDI patients exhibited lower baseline levels of serum albumin (median 30 vs. 32 g/L, *P* = 0.047). Leukocyte counts, serum creatine levels, and blood glucose levels were comparable among the two groups. There also were no apparent differences of tube or formula types found between two groups. Compared with CDN patients, CDI patients were more likely to receive more carbapenems (83.3% vs 61.4%, *P* = 0.068) and less metronidazole (27.8% vs. 63.2%, *P* = 0.037). The two groups had similar distributions of PPI usage and amount of antibiotics received.

**Table 1:** Characteristics of *C. difficile* infection (CDI) and *C. difficile*-negative (CDN) patients with enteral nutrition (EN) admitted to ICU

Characteristics	CDI (*n* = 18)	CDN (*n* = 145)	*P* value
	*N* (%)/median (IQR)	*N* (%)/median (IQR)	
**Demographics**			
Female	6 (33.3)	62 (42.8)	0.444
Age, years	66 (57.75–73.75)	48 (37–64)	0.021*
Duration of hospitalization (days)	30 (27.75–50.75)	20 (14–32)	0.001**
In-hospital mortality	1 (5.6)	17 (11.7)	0.697
Surgical intervention in previous 6 months	5 (27.8)	32 (22.1)	0.805
**Clinical features**			
CCI	2.5 (1–5)	2 (1–3)	0.015*
Comorbidities by category			
Gastrointestinal disease	0 (0)	18 (12.4)	0.236
Liver disease	5 (27.8)	52 (35.9)	0.498
Gall bladder, biliary tract, or pancreatic disease	13 (72.2)	110 (75.9)	0.962
Respiratory disease	3 (16.7)	26 (17.9)	1.000
Cardiovascular disease	5 (27.8)	55 (37.9)	0.400
Renal disease	5 (27.8)	18 (12.4)	0.159
Neurologic disease	0 (0)	5 (3.4)	0.940
Malignancy	1 (5.6)	7 (4.8)	1.000
Hematologic or immunologic disorders	2 (11.1)	20 (13.8)	1.000
Metabolic disorders	9 (50)	91 (62.8)	0.294
Clinical common underlying disease			
Diabetes	4 (22.2)	31 (21.4)	1.000
Fatty liver	4 (22.2)	41 (28.3)	0.588
Hypertension	2 (11.1)	42 (29)	0.184
History of cerebral infarction	4 (22.2)	5 (3.4)	0.006**
Laboratory results			
Leukocyte count (× 10^9^/L)	11.44 (8.32–13.86)	11.63 (8.53–15.31)	0.781
Serum albumin (g/L)	30 (26–33.5)	32 (28–36)	0.047*
Serum creatinine (μmol/L)	70 (54.8–177)	71 (55–134)	0.470
Blood glucose (mmol/L)	11.64 (9.71–13.27)	9.57 (6.95–12.98)	0.405
**In-hospital medications**			
Tube type			
Nasogastric	5 (27.8)	29 (20)	0.320
Nasojejunal	12 (66.7)	114 (78.6)	
Others^#^	1 (5.6)	2 (1.4)	
Formula use			
Semi-elemental	14 (77.8)	107 (73.8)	0.937
Polymeric	4 (22.2)	38 (26.2)	
PPI use	16 (88.9)	138 (95.2)	0.580
Antibiotics			
3rd and 4th generation cephalosporins	9 (50)	97 (66.9)	0.156
Carbapenems	15 (83.3)	89 (61.4)	0.068
Metronidazole	5 (27.8)	78 (63.2)	0.037^*^
Vancomycin	6 (33.3)	49 (34)	0.953
Fluoroquinolones	5 (27.8)	26 (17.9)	0.493
Linezolid	2 (11.1)	20 (13.8)	1.000
Aminoglycoside	1 (5.6)	10 (6.9)	1.000
Tetracycline	2 (11.1)	9 (6.2)	0.776
Antifungal agents	3 (16.7)	26 (17.9)	1.000
Antiviral drugs	0 (0)	8 (5.6)	0.654
Number of antibiotics received			
1~2	9 (50)	74 (51)	0.895
3~4	7 (38.9)	50 (34.5)	
≥ 5	2 (11.1)	21 (14.5)	

Numerical data are shown as median (interquartile range), and categorical data are described as frequency (percentage)

*Abbreviations*: *CCI* Charlson comorbidities index, *PPI* proton pump inhibitor, *IQR* interquartile range

^#^There was 1 CDI patient and 1 CDN patient receiving EN with jejunostomy tube, and 1 CDN patient with nasoduodenal tube

*P < 0.05; **P < 0.01

Finally, we assessed the potential risk factors for CDI in patients with EN using a multivariable logistic regression analysis, calculated as odds ratios (ORs) and 95% confidence intervals (CIs) (Table 2). In the final model, history of cerebral infarction was identified as a significant risk factor associated with CDI among patients with EN (OR, 9.759; 95% CI, 2.140–44.498), while prior therapy with metronidazole played a protective role (OR, 0.287; 95% CI, 0.091–0.902).

**Table 2:** Multivariate analysis of variables associated with *C. difficile* infection (CDI) in patients with enteral nutrition (EN) admitted to ICU

Model	Variables	Multivariable analysis	
		OR (95% CI)	*P* value
Initial model	Age, years	1.020 (0.960–1.083)	0.528
	Duration of hospitalization (days)	1.011 (0.992–1.030)	0.262
	Surgical intervention in previous 6 months	0.798 (0.201–3.179)	0.749
	CCI	1.066 (0.645–1.762)	0.803
	Metabolic disorders	1.047 (0.292–3.750)	0.944
	History of cerebral infarction	5.049 (0.978–26.071)	0.053
	Leukocyte count (×10^9^ /L)	1.010 (0.918–1.111)	0.836
	Serum albumin (g/L)	0.977 (0.883–1.080)	0.649
	PPI use	0.728 (0.102–5.207)	0.752
	Carbapenems	2.532 (0.582–11.014)	0.215
	Metronidazole	0.321 (0.091–1.135)	0.078
Final model	History of cerebral infarction	9.759 (2.140–44.498)	0.003^**^
	Metronidazole	0.287 (0.091–0.902)	0.033^*^

*Abbreviations*: *OR* odds ratios, *CI* confidence interval, *CCI* Charlson comorbidities index, *PPI* proton pump inhibitor

*P < 0.05; ** *P*< 0.01

### Characteristics of intestinal microbiota in CDI

**Fig. 2:**
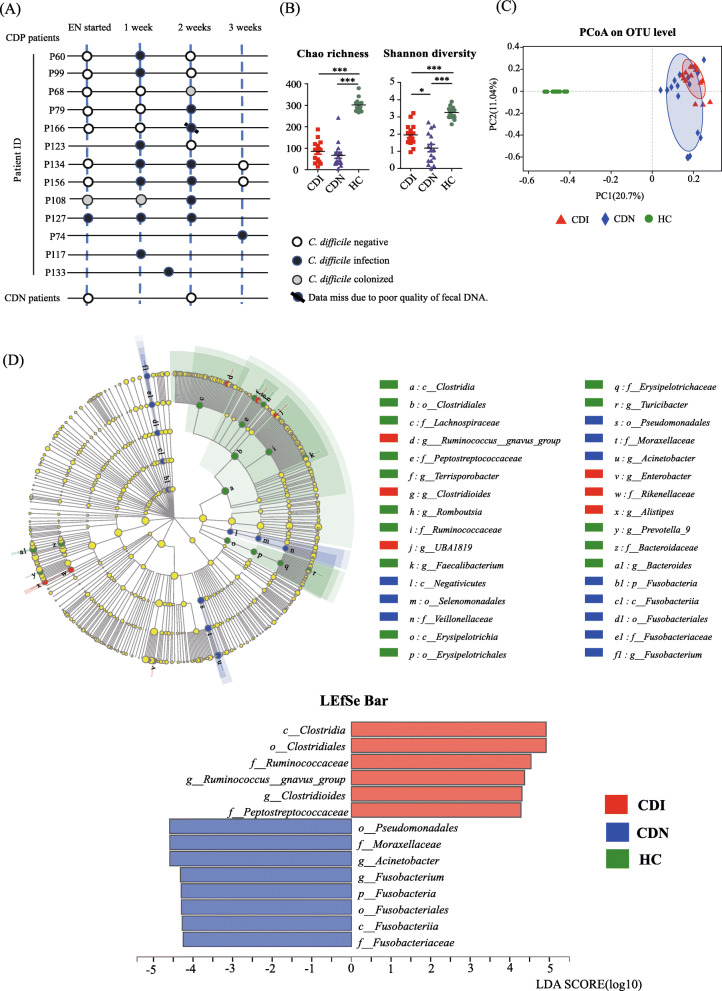
Different distribution of intestinal microbiota in CDI and CDN patients and healthy controls (HCs). **a** Fecal samples from CDP and CDN patients were collected at the indicated times and tested for the presence of *C. difficile*. White, black, and gray circles represent *C. difficle* negative, *C. difficile* infection, and *C. difficle* colonization, respectively. **b** Student’s *t* test shows differences in the indices of microbial richness and diversity between CDI (*n* = 15), CDN (*n* = 16), and HC (*n* = 12) samples. The data represent the mean value and standard error of each group. *P < 0.05; ***P < 0.001. **c** Principal coordinates analysis for CDI, CDN, and HC sample groups, with plots based on the Bray–Curtis distance. The horizontal and vertical axes represent 20.7% and 11.04% of the inter-sample variations, respectively. Each point represents a sample, and the colors represent different groups. **d** Linear discriminant analysis effect size was used to identify essential differences in abundance between CDI, CDN, and HC groups from phylum to genus. Only taxa with a significant LDA threshold value of > 4 are shown. Different-colored regions represent different groups. Circles indicate phylogenetic levels from phylum to genus. The diameter of each circle is proportional to the abundance of the group

We analyzed the microbial makeup of feces collected from 13 CDP (12 CDI and 1 CDC) and 16 CDN patients and 12 healthy controls (HCs; Fig. 2a). The CDI and CDN groups had comparable demographics, clinical features, and in-hospital medication (Table S3). Among the group, two CDI patients were detected as *C. difficile* positive at the start of EN (P108, initially presenting a negative culture result, was finally identified as positive by use of 16S gene sequencing). The CDI sample from P166 was excluded because of poor fecal DNA quality. In total, 15 CDI feces were collected. Next, we assessed the microbial composition of CDI (*n* = 15, marked as black circles in Fig. 2a), CDN (*n* = 16, collected 2 weeks after EN started from CDN patients as controls), and HC (*n* = 12) stool samples to determine defining features of intestinal microbiota among the patients.

Compared with those in the HC group, microbial richness and diversity decreased significantly in the CDI and CDN stool samples, as demonstrated by Chao richness and Shannon diversity indices (*P* < 0.001) (Fig. 2b). Interestingly, microbial diversity of the CDI group was higher than that of the CDN group (*P* < 0.05, Fig. 2b). In addition, the PCoA plot of Bray–Curtis distance revealed that CDI, CDN, and HC samples could all be different subject clusters (Adonis analysis: HC vs. CDI, CDN: *R*^2^ = 0.2414, *P* = 0.001; CDI vs. CDN: *R*^2^ = 0.0670, *P* = 0.009, Fig. 2c). The distribution of dominant bacterial phyla, families, and genera in each group is listed in Figure S1. We then used a logarithmic LDA score cutoff of 4.0 to identify important taxonomic differences among the groups (Fig. 2d). Compared with HCs, we observed significant decreases in the abundance of Bacteroidaceae (*Bacteroides*), *Prevotella_9*, Lachnospiraceae, and Ruminococcaceae (*Faecalibacterium*) in CDI and CDN samples. Concerning the differences between the CDI and CDN groups, we found that the relative abundances of *Clostridioides*, Ruminococcaceae, and *Ruminococcus_gnavus_group* were significantly higher, while *Acinetobacter* and *Fusobacterium* had lower relative abundances in CDI samples.

### Intestinal microbiota dynamics in CDI patients with EN

**Fig. 3:**
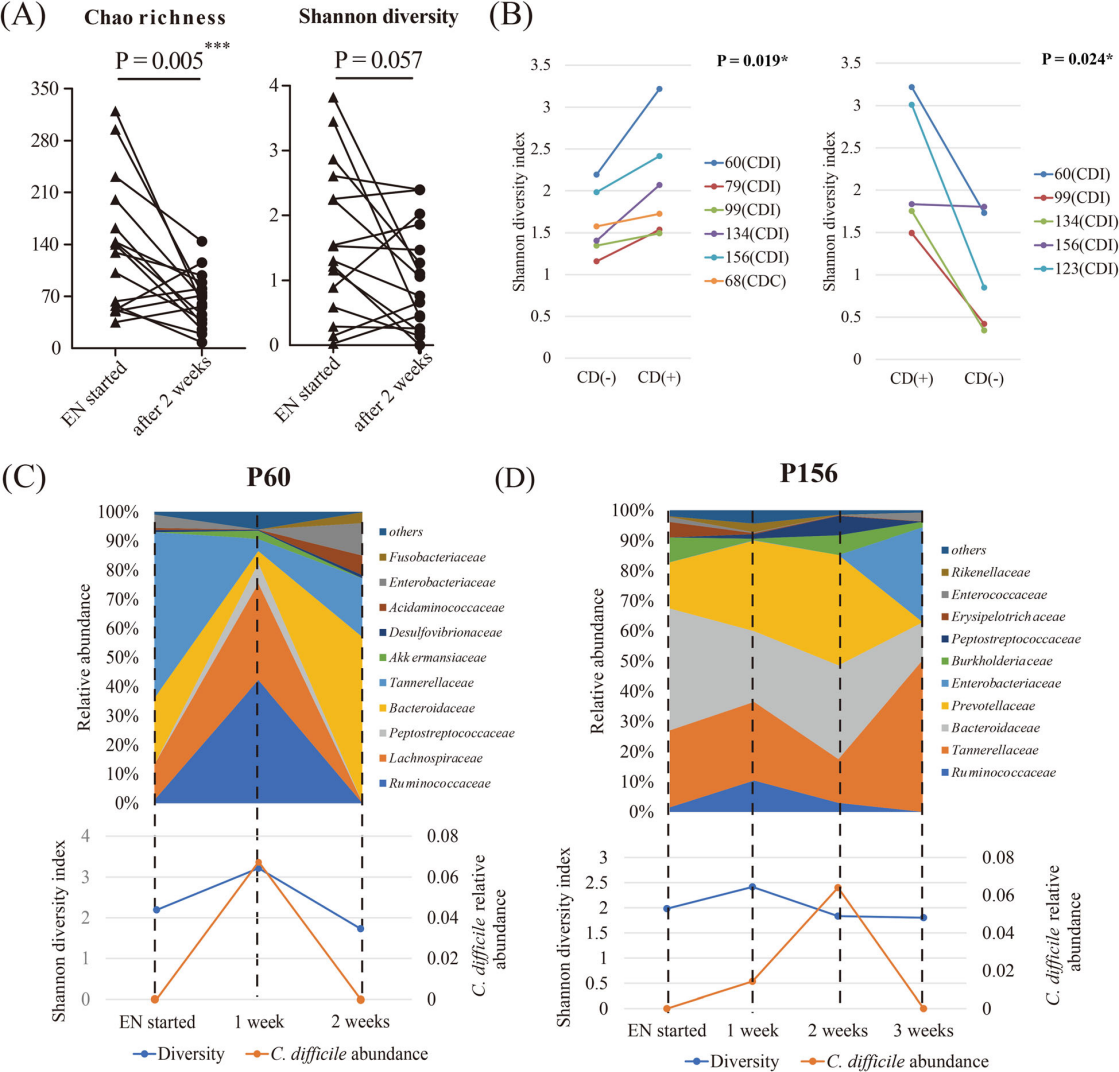
Alterations in the composition of gut microbiota in the presence of *C. difficile*. **a** Alterations in the microbial richness and diversity in CDN patients (*n* = 16) at the start of EN and 2 weeks after receiving EN. **b** Changes in microbial diversity of *C. difficile*-positive patients from *C. difficile* negative to positive (left) or *C. difficile* positive to negative (right). Statistical significance in **a** and **b** was determined using paired *t* tests. **c**, **d** Intestinal microbiota dynamics in P60 and P156. Changes in microbial composition at the family level are illustrated on the above axis, corresponding to the alterations in the *C. difficile* load (right) and microbial diversity (left) on the same timeline shown below

By comparing the microbiota of CDN patients at the start of EN and 2 weeks later, we observed that the microbial richness (*P* = 0.005) and diversity (*P* = 0.057) were declining during EN (Fig. 3a). However, in CDI patients, the trends were totally different. The overall changes in intestinal microbiota of CDI patients are shown in Figures S2 and 3. For CDI patients, the diversity increased significantly after *C. difficile* emerged (*P* = 0.019, Fig. 3b), and this trend subsequently disappeared when *C. difficile* had cleared (*P* = 0.027, Fig. 3b).These results indicate that the presence of *C. difficile* might cause a transient increase in the diversity of gut microbiota. Moreover, accompanied by the alteration of microbial diversity, the composition of microbiota also changed. For example, in P60, the relative abundance of Lachnospiraceae and Ruminococcaceae increased when *C. difficile* emerged, but decreased when *C. difficile* disappeared, in accordance with the changes in microbial diversity (Fig. 3c). Similarly, this consistent trend in changes in microbial diversity and relative abundance of Lachnospiraceae or Ruminococcaceae was found in all *C. difficile* positive (CDP) patients (Figure S2), except in P68, whose microbial diversity was accompanied by the emergence of *Phascolarctobacterium*, a short-chain fatty acid (SCFA)-producing genus (Figure S2) [15]. To further investigate the effect of this phenomenon, we focused on such patients who remained *C. difficile* positive for at least 2 weeks. As shown in Fig. 3d for P156, the diversity of gut microbiome very clearly decreased in accordance with the increase of *C. difficile* load. The same is true with respect to Lachnospiraceae and Ruminococcaceae loads.

### Role of *Bacteroides* in CDI during EN

Besides demographics, clinical features, and in-hospital medications, we speculated whether the gastrointestinal microbiome at the beginning of EN could also affect the outcomes for CDI in patients with EN. Seven CDP patients (six CDI and one CDC) without *C. difficile* colonization at the onset of EN were included in the next comparison with CDN patients.

Although there were no significant differences in microbial richness or diversity at the beginning of EN between CDN and CDP patients (Figure S3a), their compositions of microbiota in fact differed significantly (Adonis analysis, *R*^2^ = 0.0896, *P* = 0.019) (Figure S3b). We found a series of bacterial taxa, including *Bacteroides*, *Escherichia–Shigella*, *Serratia*, *Ralstonia*, and *Anaerostipes*, with distinct abundances between CDP and CDN patients (Figures S3c, 4a)*.* Given that metronidazole, a commonly used antibiotic in the clinic, could reduce the amount of *Bacteroides* in the human intestine, we excluded patients who received prior metronidazole treatment within 3 days before starting EN to avoid the interference of medications [12]. Nonetheless, the difference in abundance of *Bacteroides* was still significant between the two groups (*P* = 0.011, Figure S3d). It was suggested that, even at the earliest initiation of EN, CDP patients had already presented different structures of gut microbiota, namely featuring higher proportions of *Bacteroides*.

**Fig. 4:**
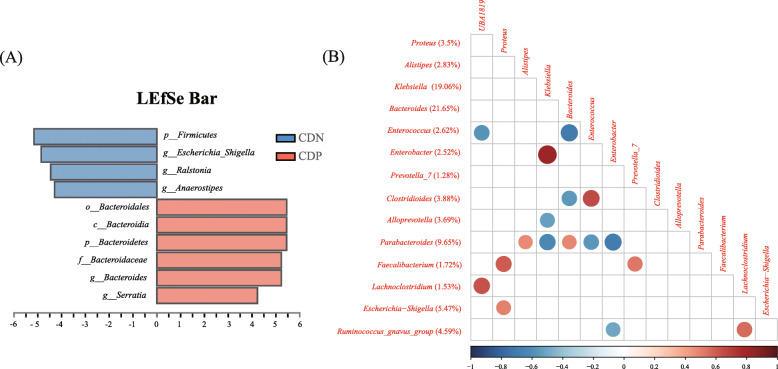
Role of *Bacteroides* in CDI. **a** Linear discriminant analysis effect size was used to compare the composition of intestinal microbiota among CDP (*n* = 7) and CDN (*n* = 16) patients at the beginning of EN. Only taxa with a significant LDA threshold value of > 4 are shown. **b** Correlation matrix of the relative abundance of predominant genera in all *C. difficile*-positive samples (*n* = 18). The average percentage of each genus among all samples is shown to the left of the name of the genus. Only significant correlations are shown (*P* < 0.05). The size and color of the circles reflect the degree and direction of the correlation, respectively. Correlations were all tested using Spearman’s correlation test

To clarify the role of *Bacteroides* in CDI, we observed that *Bacteroides* loads tended to decrease after infection of *C. difficile* (40.64% vs. 23.09%, *P* = 0.093), while the abundance of *Bacteroides* remained stable for CDN patients during EN (10.51% vs. 8.53%, *P* = 0.451) (Figure S3e). Subsequently, we conducted a correlation analysis among all feces positive for *C. difficile*. Interestingly, the relative abundance of *Clostridioides* was significantly negatively correlated with that of *Bacteroides* (*R* = − 0.58, *P* = 0.016), but also significantly positively correlated with that of *Enterococcus* (*R* = 0.66, *P* = 0.002) (Fig. 4b). These correlations were verified using quantitative PCR analysis (Figure S3f), which together indicates a possible mutual inhibitory relationship between *Bacteroides* and *C. difficile* in CDI.

## Discussion

CDI has emerged as one of the most threatening human health problems found globally throughout healthcare facilities, especially in ICUs [14]. The prevalence of CDI among ICU patients, measured at approximately 2%, is reported to be significantly higher than the prevalence of CDI among general ward patients, as measured at about 0.9% [16]. In the present study, we investigated patients admitted to ICUs receiving EN therapy for at least 1 week. The prevalence of CDI reached 10.71%, which is much higher than the 0.4–4% estimated among ICU patients in European countries, and also higher than the 4.12% we reported in a previous study of ICU patients at our institution [10, 14, 17]. It is certainly possible that our reporting of CDI prevalence is attributed to more frequent exposure to *C. difficile* spores through the feeding tube, and/or to heavy usage of antibiotics or PPIs. We studied ICU patients receiving EN and revealed that patients presenting CDI were generally older, had longer hospital stays, and presented higher CCI scores and lower serum albumin levels. Despite other frequent risk factors, we noticed that history of cerebral infarction was strongly associated with CDI occurrence, which may be due to old age, to low microbial diversity, or to the possibility of prior long-term healthcare institution exposure [18]. Metronidazole is commonly used to treat CDI and, consistent with many other studies, was recognized as a protective factor, emphasizing its importance in the prevention and treatment of CDI [10, 17, 19, 20]. PPIs and the number of antibiotics used are classical risk factors for CDI, but they do not vary significantly between the two groups [8, 10]. This occurrence may be due to extremely high CDI prevalence in this population. Overall, these results collectively enhance the epidemiological data for CDI and emphasize the importance for further attention to ICU patients receiving EN.

EN, along with the prophylactic use of antibiotics and PPIs, is likely to be accompanied by a disruption or remodeling of the gut microbiota, which plays an essential role in the occurrence and development of CDI [9, 21]. Compared with those from HCs, samples from our CDI and CDN patients showed a significant decrease in microbial richness and diversity, with lower abundance of Bacteroidaceae (*Bacteroides*), Lachnospiraceae, and Ruminococcaceae (*Faecalibacterium*), all of which are necessary to maintain intestinal homeostasis [22–25]. Over the course of the 2 weeks of EN, the microbial richness and diversity continued to decline. *Enterococcus* species, which are highly associated with nosocomial infection in ICUs, increased substantially in the samples studied and exhibited a positive correlation with presence of *C. difficile* [26]. All these results collectively indicate that ICU patients with EN have a fragile gut microbiota, and the course of EN treatment further disrupts the microbiota. This adverse condition may be triggered not only by the heavy use of antibiotics and PPIs, but also by the distinct diets in EN. Although EN formulas contain essential nutrients for patients, they usually have less proportion of fiber than normal diets, which can be fermented by colonic microbiota to produce regulators of colonic epithelial proliferation to protect against gut pathogens [9]. CDN patients were perhaps increasingly susceptible to *C. difficile* due to their equally poor microbial structures, even though they had not been exposed to *C. difficile* spores. Overall, the poor intestinal microbiota of ICU patients receiving EN may facilitate *C. difficile* expansion and make patients more vulnerable to CDI.

Previous studies have often described microbiota characteristics of CDI patients in comparison to those of HCs or *C. difficile*-negative patients with diarrhea [27–30]. However, it is difficult to clarify precisely to what these microbial changes should be attributed, whether it be diarrhea, complex clinical management, or *C. difficile* itself. Patients with EN receiving both consistent diets and clinical management could represent a suitable group in which to observe the microbial features in CDI. By comparing feces between CDI and CDN patients, we found a surprising increase in the microbial diversity in CDI samples, along with higher abundance of Ruminococcaceae and *R. gnavus_group* (Lachnospiraceae family). We further analyzed the dynamics of the intestinal microbiota throughout pathogenesis of CDI. Our analysis revealed that the presence of *C. difficile* may cause a transient increase in microbial diversity, with a consistent change in the abundance of Ruminococcaceae and Lachnospiraceae families or other SCFA-producing bacteria. Ruminococcaceae and Lachnospiraceae families are usually recognized as protective microbes against CDI, depending on their ability to produce SCFAs and secondary bile acids [25, 31]. SCFAs, especially butyrate, can enhance colonic defense barriers by secreting antimicrobial peptides, and secondary bile acids can directly restrain *C. difficile* germination or vegetative growth [32, 33]. Accordingly, although the accurate mechanisms for these noteworthy microbial alterations remain unclear, we speculate that the mechanisms might be due to protective reactions against *C. difficile* overgrowth. The opposite relationship between microbial diversity and *C. difficile* load observed in several patients with long duration of CDI also supports this hypothesis. Vincent et al. [34] described a similar response to *C. difficile* colonization, proposing an increase of beneficial bacterial taxa in the gut, including Clostridiales Family XI Incertae Sedis, *Clostridium* and *Eubacterium*. In sum, these findings suggest that a potential protective microbial reaction may appear in response to the emergence of *C. difficile*, thus enriching our understanding of how hosts respond to CDI.

Given that EN therapy increased the risk of CDI, we attempted to evaluate relevant risk factors for CDI within the intestinal microbiota. We observed a greater proportion of *Bacteroides* in CDP patients before EN therapy, suggesting that *Bacteroides* promotes colonization of *C. difficile*. However, we also detected an inhibitory relationship between *Bacteroides* and *C. difficile*, as demonstrated by their negatively correlated abundances. These contradictory results raise interesting questions for us: What is the role of *Bacteroides* in the development of CDI? Does *Bacteroides* serve as a risk factor or a defender? Previous investigations also revealed similarly inconsistent conclusions. Based on mouse models, Li et al. [35] demonstrated that *Bacteroides* was positively correlated with *C. difficile* loads, while Sangster et al. [36] found the opposite to be true in clinical CDI samples. We know for certain that *Bacteroides* species interact with *C. difficile* in different means. *Bacteroides fragilis*, *Bacteroides ovatus*, and *Bacteroides vulgatus* can all protect against CDI through production of SCFAs or secondary bile acids [37, 38]. However, Ferreyra et al. [39] and Ng et al. [40] have both demonstrated that *Bacteroides thetaiotaomicron* metabolizes polysaccharides to provide *C. difficile* with a source of nutrition, such as sialic acid and succinate, and helps it proliferate in the perturbed intestine. Thus, we propose that *Bacteroides* species might play different roles in CDI at different stages of EN. Perhaps early on in EN, in response to the higher concentrations of colonic polysaccharide present in the intestine, *Bacteroides* may play a dominant role in providing substrates for *C. difficile* growth. Then, after a period of elemental diets of lower polysaccharide concentration, the function of *Bacteroides* shifts to producing SCFAs and secondary bile acids, taking precedence in protecting against CDI. Undoubtedly, the complex interactions among intestinal microbiota are one plausible reason for such divergent conclusions. Further research is necessary to clarify the detailed mechanisms by which different species of *Bacteroides* act during the course of CDI.

We believe the current study to be the first of its kind to focus on CDI in ICU patients with EN. After effectively ruling out dietary interventions and clinical management, we took a closer look at the structure of the intestinal microbiota, the results of which provide new insights into the association between gut pathogens and symbiotic microflora. However, we recognize several limitations to our work. First, all participants were from a single health center, meaning that the results may not be applicable across all healthcare institutions. Secondly, we were limited to selecting otherwise healthy individuals to serve as our control population to contrast the vulnerable microbial environments as revealed in patients with EN. In order to specify the exact impact of EN on intestinal microbiota, selecting patients already in the ICU but without having undergone any EN therapy might have been a more appropriate study design approach. Thirdly, our observations on risk factors, microbial characteristics, and dynamics were limited by a small sample size. The risk factors and microbial characteristics in this cohort necessitate larger study populations in order to draw larger conclusions. Finally, to better understand the interaction between *C. difficile* and the intestinal microbiota, further studies with more expansive experimental results are required. One possible route of research may incorporate metabolomic applications of the gut microbiota.

## Conclusion

The overall incidence of CDI reached 10.7%. History of cerebral infarction significantly increased the risk of CDI, while treatment with metronidazole was a protective factor. Patients with EN exhibited fragile intestinal environments, making it more vulnerable to CDI. When CDI occurred, a potentially protective alteration of gut microbiota appeared, exhibiting increased microbial diversity and abundance of some beneficial bacterial taxa. *Bacteroides* play vital yet possibly different roles in CDI formation and development. In summary, our study provides useful epidemiological data for CDI development in patients with EN and enhances our understanding of the interaction between *C. difficile* and intestinal microbiomes.

## Supplementary information

**Additional file 1 : Table S1.** Primers used in this study.

**Additional file 2 : Table S2.** Detection of *C. difficile* and its toxins in *C. difficile* positive patients. a. Y, yes; b. N. no.

**Additional file 3 : Table S3.** Detailed information on participants selected for microbiota analysis. a. M, male; b. F, female.

**Additional file 4 : Figure S1.** Microbial composition in CDI, CDN and HC samples. (A) Average relative proportions of phyla, families, and genera in each group. (B) Wilcoxon rank sum test was used to compare relative abundances at the phylum, family, and genus levels among groups. Significant differences of CDI vs. CDN, CDN vs. HC, and CDI vs. HC are illustrated as “*”, “#”, and “$”, respectively. *#$ *P* < 0.05; ** ## $$ *P* < 0.01; *** ### $$$ *P* < 0.001.

**Additional file 5 : Figure S2.** Intestinal microbiota dynamics in *C. difficile* positive (CDP) patients. For each panel, changes in microbial composition at the family level are illustrated on the above axis, corresponding to the alterations in the *C. difficile* load (right) and microbial diversity (left) on the same timeline shown below.

**Additional file 6 : Figure S3.** Role of *Bacteroides* in CDI (A) and (B) The comparation in the composition of intestinal microbiota between CDP (*n* = 7) and CDN (*n* = 16) patients at the beginning of EN. (A) Differences of microbial richness and diversity, tested using Student’s t-tests. (B) Principal coordinates analysis plots based on the Bray-Curtis distance. (C) and (D) Wilcoxon rank sum tests were performed to analyze the differences between 7 CDP and 16 CDN patients (C) or those excluded because they were treated with metronidazole within 3 days of onset of EN (D). (E) Relative abundance of *Bacteroides* genus from the onset of EN to the first presence of *C. difficile* for CDP patients (*n* = 6; left), or from the onset of EN to 2 weeks later for CDN patients (n = 16; right). Differences were evaluated by Wilcoxon signed-rank test. (F) Correlation between the relative abundance of *Bacteroides* or *Enterococcus* and *C. difficile* calculated using qPCR, and analyzed using a Spearman’s correlation test. *P < 0.05, **P < 0.01.

## Data Availability

The datasets used and/or analyzed in the current study are available by the corresponding author upon reasonable request.

## Abbreviations

EN: Enteral nutrition
ICU: Intensive care unit
CDI: *C. difficile* infection
PPI: Proton pump inhibitor
CDC: *C. difficile* colonization
CDP: *C. difficile* positive
CDN: *C. difficile* negative
ELFA: Enzyme-linked fluorescence assay
OTU: Operational taxonomic unit
CCI: Charlson comorbidity index
OR: Odds ratio
CI: Confidence interval
PCoA: Principal coordinates analysis
LEfSe: Linear discriminant analysis effect size
LDA: Linear discriminant analysis
IQR: Interquartile range
SCFA: Short-chain fatty acid

## References

[1] Abt MC, McKenney PT, Pamer EG (2016). *Clostridium difficile* colitis: pathogenesis and host defence. Nat Rev Microbiol.

[2] Lim SC, Knight DR, Riley TV. Clostridium difficile and One Health. Clin Microbiol Infect. 2020;26(7):857–863. 10.1016/j.cmi.2019.10.023 31682985

[3] Kuy S (2016). Increasing incidence of and increased mortality associated with *Clostridium difficile*-associated megacolon. JAMA Surg.

[4] Samarkos M, Mastrogianni E, Kampouropoulou O (2018). The role of gut microbiota in *Clostridium difficile* infection. Eur J Intern Med.

[5] Dubberke ER (2007). *Clostridium difficile*--associated disease in a setting of endemicity: identification of novel risk factors. Clin Infect Dis.

[6] Bliss DZ (1998). Acquisition of *Clostridium difficile* and *Clostridium difficile*-associated diarrhea in hospitalized patients receiving tube feeding. Ann Intern Med.

[7] Balsells E (2019). Global burden of infections: a systematic review and meta-analysis. J Glob Health.

[8] Barletta JF, Sclar DA (2014). Proton pump inhibitors increase the risk for hospital-acquired *Clostridium difficile* infection in critically ill patients. Crit Care.

[9] O'Keefe SJ. Tube feeding, the microbiota, and Clostridium difficile infection. World J Gastroenterol. 2010;16(2):139–42. 10.3748/wjg.v16.i2.139 PMC2806551 20066732

[10] Cui, Y., et al., Risk factors for Clostridioides difficile infection and colonization among patients admitted to an intensive care unit in Shanghai, China*,* BMC Infect Dis, 2019. 19(1): p. 961. 10.1186/s12879-019-4603-1 PMC6849324 31711425

[11] Theriot CM, Young VB (2015). Interactions between the gastrointestinal microbiome and *Clostridium difficile*. Annu Rev Microbiol.

[12] Bobo LD, Dubberke ER, Kollef M (2011). *Clostridium difficile* in the ICU: the struggle continues. Chest.

[13] DDebast SB, Bauer MP, Kuijper EJ. European Society of Clinical Microbiology and Infectious Diseases: update of the treatment guidance document for *Clostridium difficile* infection. Clin Microbiol Infect. 2014;20(Suppl 2):1–26. 10.1111/1469-0691.12418 24118601

[14] Prechter F (2017). Sleeping with the enemy: *Clostridium difficile* infection in the intensive care unit. Crit Care.

[15] Wu F (2017). Phascolarctobacterium faecium abundant colonization in human gastrointestinal tract. Exp Ther Med.

[16] Lucado J, Gould C, Elixhauser A (2006). *Clostridium difficile* infections (CDI) in hospital stays, 2009: statistical brief# 124.

[17] Manthey CF, et al. Initial therapy affects duration of diarrhoea in critically ill patients with Clostridioides difficile infection (CDI). Crit Care. 2019;23(1):399. 10.1186/s13054-019-2648-6 PMC6902451 31815650

[18] Wang W (2018). The characteristics analysis of intestinal microecology on cerebral infarction patients and its correlation with apolipoprotein E. Medicine (Baltimore).

[19] Guh AY (2017). Risk factors for community-associated *Clostridium difficile* infection in adults: a case-control study. Open Forum Infect Dis.

[20] Teasley DG (1983). Prospective randomised trial of metronidazole versus vancomycin for Clostridium-difficile-associated diarrhoea and colitis. Lancet (London).

[21] Whelan K, Schneider SM (2011). Mechanisms, prevention, and management of diarrhea in enteral nutrition. Curr Opin Gastroenterol.

[22] Bäckhed F (2005). Host-bacterial mutualism in the human intestine. Science (New York).

[23] Wexler HM (2007). Bacteroides: the good, the bad, and the nitty-gritty. Clin Microbiol Rev.

[24] Atarashi K (2013). Treg induction by a rationally selected mixture of Clostridia strains from the human microbiota. Nature.

[25] Lee YJ (2017). Protective factors in the intestinal microbiome against *Clostridium difficile* infection in recipients of allogeneic hematopoietic stem cell transplantation. J Infect Dis.

[26] Freedberg DE (2018). Pathogen colonization of the gastrointestinal microbiome at intensive care unit admission and risk for subsequent death or infection. Intensive Care Med.

[27] Zhang L (2015). Insight into alteration of gut microbiota in *Clostridium difficile* infection and asymptomatic *C. difficile* colonization. Anaerobe.

[28] Schubert AM (2014). Microbiome data distinguish patients with *Clostridium difficile* infection and non-*C. difficile*-associated diarrhea from healthy controls. mBio.

[29] Gu S (2016). Identification of key taxa that favor intestinal colonization of *Clostridium difficile* in an adult Chinese population. Microbes Infect.

[30] Antharam VC (2013). Intestinal dysbiosis and depletion of butyrogenic bacteria in *Clostridium difficile* infection and nosocomial diarrhea. J Clin Microbiol.

[31] Crobach MJT, et al. Understanding Clostridium difficile Colonization. Clin Microbiol Rev. 2018;31(2):e00021–17. 10.1128/CMR.00021-17 PMC5967689 29540433

[32] Morrison DJ, Preston T (2016). Formation of short chain fatty acids by the gut microbiota and their impact on human metabolism. Gut Microbes.

[33] Buffie CG (2015). Precision microbiome reconstitution restores bile acid mediated resistance to *Clostridium difficile*. Nature.

[34] Vincent C (2016). Bloom and bust: intestinal microbiota dynamics in response to hospital exposures and *Clostridium difficile* colonization or infection. Microbiome.

[35] Li X (2019). Consortium of probiotics attenuates colonization of Clostridioides difficile. Front Microbiol.

[36] Sangster W, et al. Bacterial and Fungal Microbiota Changes Distinguish C. difficile Infection from Other Forms of Diarrhea: Results of a Prospective Inpatient Study. Front Microbiol. 2016;7:789. 10.3389/fmicb.2016.00789 PMC4879479 27252696

[37] Deng H (2018). Bacteroides fragilis prevents *Clostridium difficile* infection in a mouse model by restoring gut barrier and microbiome regulation. Front Microbiol.

[38] Mullish BH (2019). Microbial bile salt hydrolases mediate the efficacy of faecal microbiota transplant in the treatment of recurrent infection. Gut.

[39] Ferreyra JA (2014). Gut microbiota-produced succinate promotes C. difficile infection after antibiotic treatment or motility disturbance. Cell Host Microbe.

[40] Ng KM (2013). Microbiota-liberated host sugars facilitate post-antibiotic expansion of enteric pathogens. Nature.

