# Supplementary material for: Risk factors and intestinal microbiota: Clostridioides difficile infection in patients receiving enteral nutrition at Intensive Care Units
Source: Crit Care. 2020 Jul 13;24:426. doi: 10.1186/s13054-020-03119-7 (PMC7359293; doi:10.1186/s13054-020-03119-7)
Supplement: Supplementary file 1 — Additional file 1 : Table S1. Primers used in this study. [file 13054_2020_3119_MOESM1_ESM.docx]

| Primers | Sequences (5’→3’) |
| --- | --- |
| *gluD-*F | GGAAAAGATGTAAATGTCTTCGAGATG |
| *gluD-*R | CTGATTTACACCATTCAGCCATAGC |
| *ToxinA-*F | TTTTGATCCTATAGAATCTAACTTAGTAAC |
| *ToxinA-*R | CCACCAGCTGCAGCCATA |
| *ToxinB-*F | GTGTAGCAATGAAAGTCCAAGTTTACGC |
| *ToxinB-*R | CACTTAGCTCTTTGATTGCTGCACCT |
| qPCR |  |
| *Bacteroides*-F | GAGAGGAAGGTCCCCCAC |
| *Bacteroides*-R | CGCTACTTGGCTGGTTCAG |
| *Enterococcus*-F | ATCAGAGGGGGATAACACTT |
| *Enterococcus*-R | ACTCTCATCCTTGTTCTTCTC |
| *C.difficile* (toxin B)-F | GAAGGTGGTTCAGGTCATAC |
| *C.difficile* (toxin B)-R | CATTTTCTAAGCTTCTTAAACCTG |
| All bacteria-F | CGGTGAATACGTTCCCGG |
| All bacteria-R | TACGGCTACCTTGTTACGACTT |

**Table S1.** Primers used in this study.
