# Supplementary material for: Risk factors and intestinal microbiota: Clostridioides difficile infection in patients receiving enteral nutrition at Intensive Care Units
Source: Crit Care. 2020 Jul 13;24:426. doi: 10.1186/s13054-020-03119-7 (PMC7359293; doi:10.1186/s13054-020-03119-7)
Supplement: Supplementary file 2 — Additional file 2 : Table S2. Detection of C. difficile and its toxins in C. difficile positive patients. a. Y, yes; b. N. no. [file 13054_2020_3119_MOESM2_ESM.docx]

**Table S2.** Detection of *C. difficile* and its toxins in *C. difficile* positive patients

| Patient ID | Culture | Toxin | ELFA | Positive detection since EN treatment (days) | Diarrhea |
| --- | --- | --- | --- | --- | --- |
| P7 | Positive | A-B+ | Positive | 4 | Y^a^ |
| P17 | Positive | A-B+ | Positive | 6 | N^b^ |
| P38 | Negative | - | Positive | 9 | Y |
| P49 | Negative | - | Positive | 43 | Y |
| P60 | Negative | - | Positive | 16 | Y |
| P68 | Positive | A-B- | Negative | 15 | N |
| P69 | Negative | - | Positive | 14 | N |
| P70 | Negative | - | Positive | 1 | Y |
| P74 | Positive | A+B+ | Positive | 23 | Y |
| P79 | Positive | A+B+ | Negative | 21 | Y |
| P90 | Negative | - | Positive | 57 | N |
| P99 | Positive | A+B+ | Positive | 8 | Y |
| P108 | Negative | - | Positive | 12 | Y |
| P117 | Negative | - | Positive | 8 | Y |
| P123 | Positive | A-B+ | Negative | 12 | Y |
| P127 | Negative | - | Positive | 1 | Y |
| P133 | Positive | A+B+ | Negative | 10 | Y |
| P134 | Positive | A-B+ | Positive | 7 | Y |
| P137 | Positive | A-B+ | Negative | 48 | Y |
| P150 | Negative | - | Positive | 33 | Y |
| P156 | Negative | - | Positive | 9 | Y |
| P158 | Positive | A-B+ | Positive | 5 | N |
| P166 | Positive | A-B+ | Positive | 14 | Y |

a. Y, yes; b. N. no.
