# Supplementary material for: Risk factors and intestinal microbiota: Clostridioides difficile infection in patients receiving enteral nutrition at Intensive Care Units
Source: Crit Care. 2020 Jul 13;24:426. doi: 10.1186/s13054-020-03119-7 (PMC7359293; doi:10.1186/s13054-020-03119-7)
Supplement: Supplementary file 3 — Additional file 3 : Table S3. Detailed information on participants selected for microbiota analysis. a. M, male; b. F, female. [file 13054_2020_3119_MOESM3_ESM.docx]

**Table S3.** Detailed information on participants selected for microbiota analysis

| Group | Patient ID | age | Gender | Comorbidities | Medication |
| --- | --- | --- | --- | --- | --- |
| CDI  (n=12) | P60 | 37 | M^a^ | Acute severe pancreatitis, hypertension, gout, systemic lupus erythematosus (SLE) | cephalosporins |
|  | P74 | 73 | F^b^ | Acute biliary pancreatitis, acute cholecystitis, schistosomiasis cirrhosis, hypertension | PPIs, cephalosporins, vancomycin, metronidazole, antifungal agents |
|  | P79 | 54 | M | Acute severe pancreatitis, diabetes, personal history of cerebral infarction, psoriasis | PPIs, cephalosporins, metronidazole, carbapenems |
|  | P99 | 69 | M | Acute severe pancreatitis, sepsis | PPIs, cephalosporins, carbapenems |
|  | P108 | 62 | F | Acute severe pancreatitis, kidney stones, fatty liver | PPIs, carbapenems |
|  | P117 | 69 | F | Acute severe pancreatitis, hypertension, abdominal wall hernia, history of breast cancer, congenital sigmoid diverticulum | PPIs, carbapenems, tetracyclines, vancomycin, aminoglycoside |
|  | P123 | 85 | M | Severe pneumonia, sepsis, personal history of cerebral infarction | PPIs, cephalosporins, fluoroquinolones, vancomycin |
|  | P127 | 27 | M | Acute severe pancreatitis, hyperlipidemia, fatty liver, kidney stones | PPIs, carbapenems |
|  | P133 | 67 | M | Acute severe pancreatitis, complete right bundle branch block | PPIs, cephalosporins, fluoroquinolones, carbapenems, metronidazole |
|  | P134 | 38 | M | Acute severe pancreatitis, hyperlipidemia, fatty liver | PPIs, carbapenems |
|  | P156 | 76 | M | Spindle cell carcinoma, pneumonia | PPIs, carbapenems, vancomycin |
|  | P166 | 66 | F | Acute severe pancreatitis, sepsis, type 2 diabetes, hyperlipidemia | PPIs, carbapenems, vancomycin, antifungal agents |
| CDC  (n=1) | P68 | 67 | M | Severe pneumonia, Parkinson's disease, lacunar infarction, fatty liver | Carbapenems, vancomycin |
| CDN  (n=16) | P2 | 35 | F | Acute severe pancreatitis, hyperlipidemia, mesenteric torsion | PPIs, cephalosporins, metronidazole |
|  | P4 | 43 | M | Acute severe pancreatitis, hyperkalemia, fatty liver, thickening of the adrenals | PPIs, carbapenems, aminoglycoside, linezolid, tetracyclines |
|  | P8 | 42 | M | Acute severe pancreatitis, hypertension, gout, personal history of cerebral infarction | PPIs, cephalosporins, metronidazole, carbapenems, vancomycin, antifungal agents |
|  | P16 | 82 | M | Severe pneumonia, gastrointestinal bleeding, t-lymphoma, hypertension | PPIs, fluoroquinolones, penicillin, antifungal agents |
|  | P18 | 71 | F | Acute severe pancreatitis, hypertension | PPIs, cephalosporins, metronidazole, carbapenems, vancomycin, aminoglycoside, linezolid |
|  | P23 | 63 | M | Acute severe pancreatitis, sepsis, duodenal tumor, hypertension, chronic gastritis, pancreatic encephalopathy | PPIs, carbapenems, vancomycin, fluoroquinolones, antifungal agents |
|  | P28 | 31 | M | Acute severe pancreatitis, sepsis | PPIs, carbapenems |
|  | P35 | 79 | M | Acute severe pancreatitis, pulmonary interstitial fibrosis, hypertension, gout | PPIs, cephalosporins, metronidazole |
|  | P43 | 34 | M | Severe pneumonia, hypertension, uremia, chronic hepatitis B | PPIs, fluoroquinolones |
|  | P54 | 32 | M | Acute severe pancreatitis | PPIs, carbapenems, linezolid |
|  | P63 | 88 | M | Sepsis, cerebral infarction, atrial fibrillation, gallbladder stones, intestinal malignancies (postoperative) | PPIs, carbapenems, cephalosporins, metronidazole antifungal agents |
|  | P75 | 75 | F | Community-acquired pneumonia, chronic kidney disease stage 5, chronic heart failure, hypertension, type 2 diabetes, renal anemia | PPIs, carbapenems, antifungal agents |
|  | P101 | 68 | M | Acute severe pancreatitis | PPIs, carbapenems, metronidazole, vancomycin |
|  | P104 | 51 | F | Acute severe pancreatitis | PPIs, cephalosporins, metronidazole, carbapenems, aminoglycoside, linezolid |
|  | P129 | 44 | M | Acute severe pancreatitis, sepsis | PPIs, carbapenems, vancomycin |
|  | P131 | 60 | M | Acute severe pancreatitis, hyperlipidemia, type 2 diabetes | PPIs, cephalosporins, metronidazole |
| HCs  (n=12) | C1 | 74 | M | - | - |
|  | C2 | 64 | M | - | - |
|  | C3 | 86 | M | - | - |
|  | C4 | 52 | M | - | - |
|  | C5 | 89 | F | - | - |
|  | C6 | 90 | M | - | - |
|  | C7 | 74 | M | - | - |
|  | C8 | 89 | M | - | - |
|  | C9 | 35 | M | - | - |
|  | C10 | 39 | M | - | - |
|  | C11 | 58 | F | - | - |
|  | C12 | 33 | F | - | - |

a. M, male; b. F, female.
