# Supplementary figures and images for: Risk factors and intestinal microbiota: Clostridioides difficile infection in patients receiving enteral nutrition at Intensive Care Units
Source: Crit Care. 2020 Jul 13;24:426. doi: 10.1186/s13054-020-03119-7 (PMC7359293; doi:10.1186/s13054-020-03119-7)

(A)

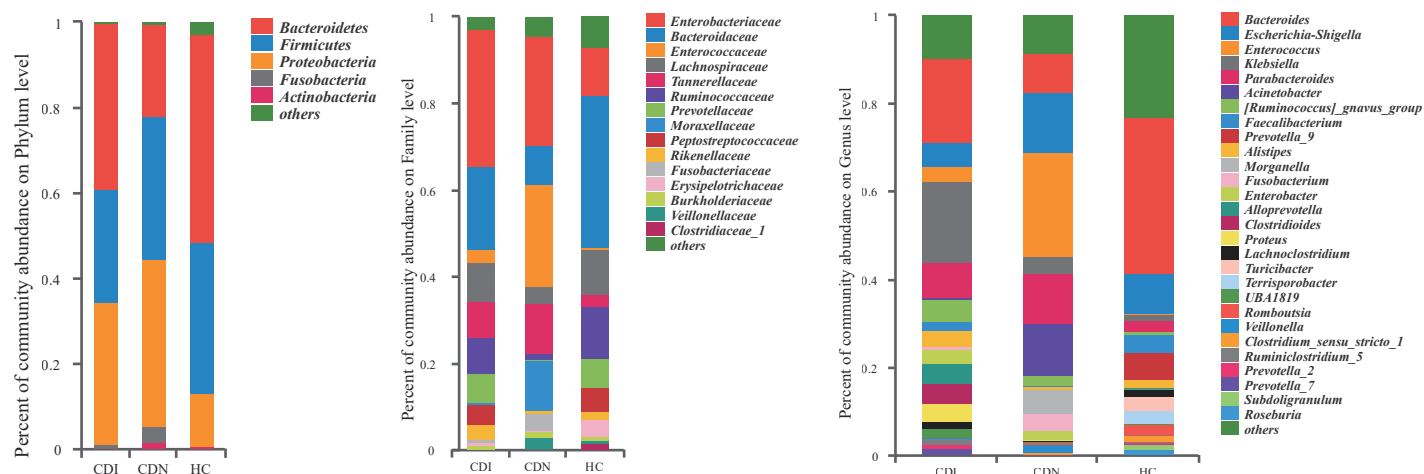

(B)

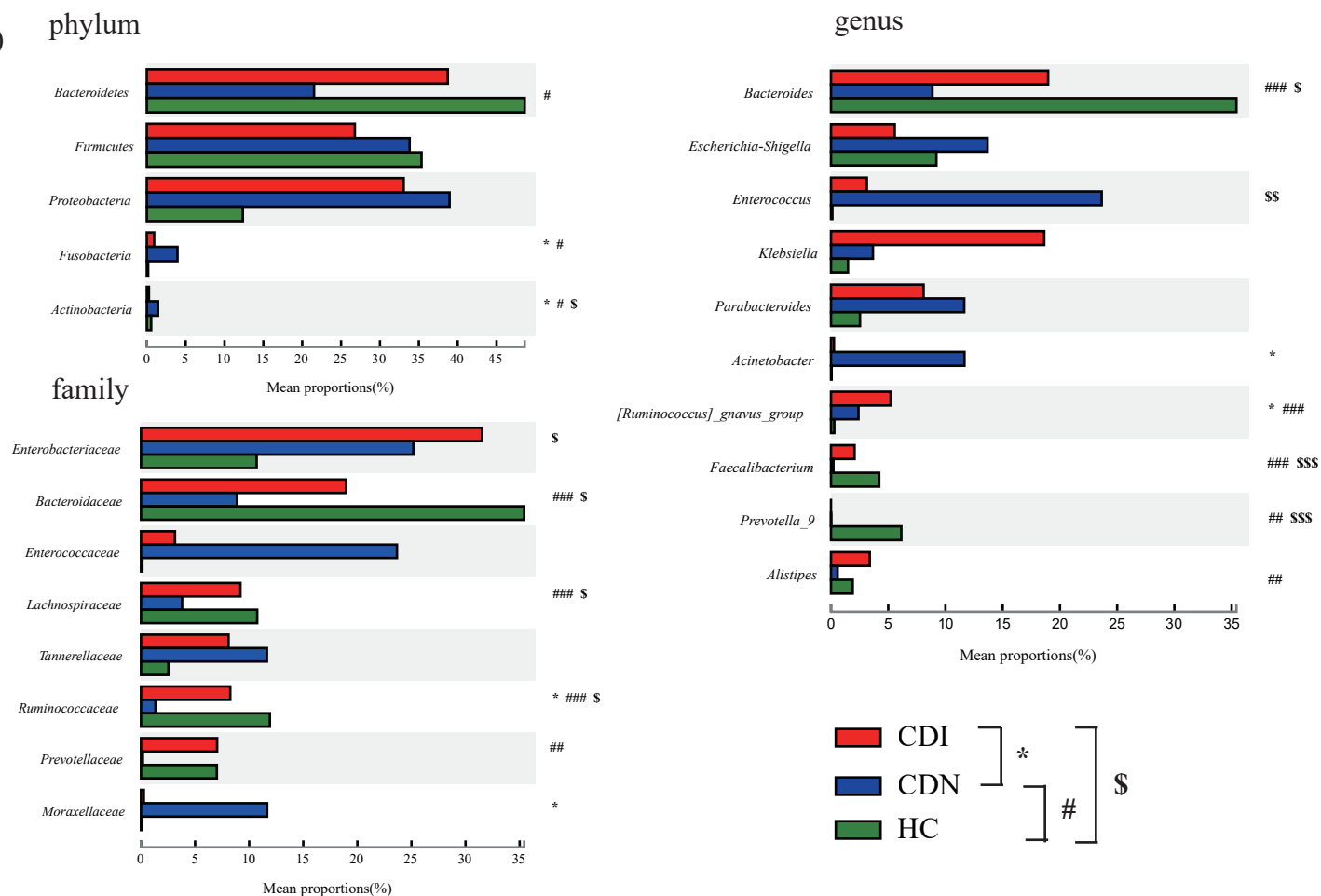

Supplement: Supplementary file 4 — Additional file 4 : Figure S1. Microbial composition in CDI, CDN and HC samples. (A) Average relative proportions of phyla, families, and genera in each group. (B) Wilcoxon rank sum test was used to compare relative abundances at the phylum, family, and genus levels among groups. Significant differences of CDI vs. CDN, CDN vs. HC, and CDI vs. HC are illustrated as “*”, “#”, and “$”, respectively. *#$ P < 0.05; ** ## $$ P < 0.01; *** ### $$$ P < 0.001. [file 13054_2020_3119_MOESM4_ESM.pdf]

Patient 68 (CDC)

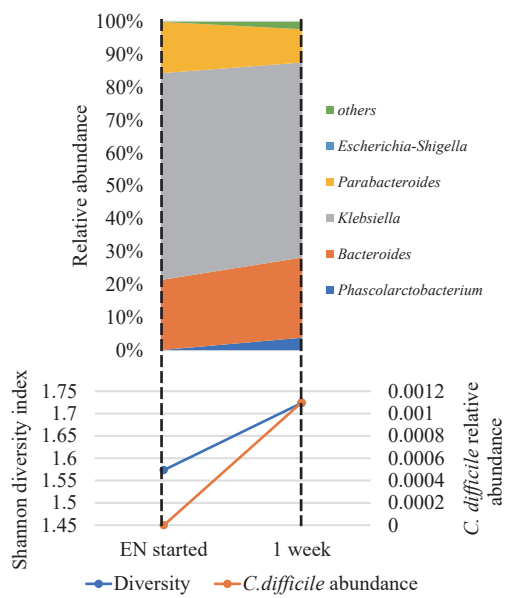

Patient 79 (CDI)

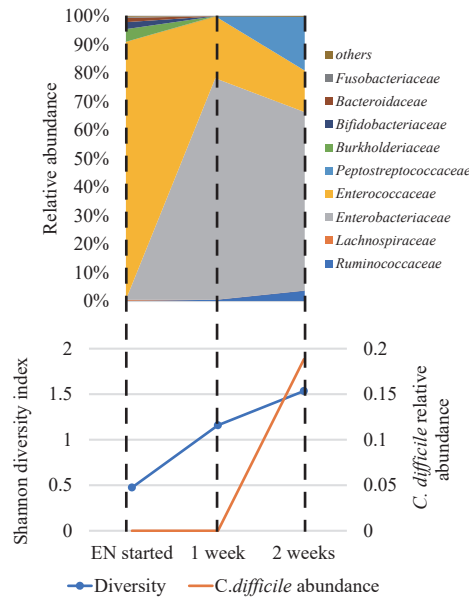

Patient 99 (CDI)

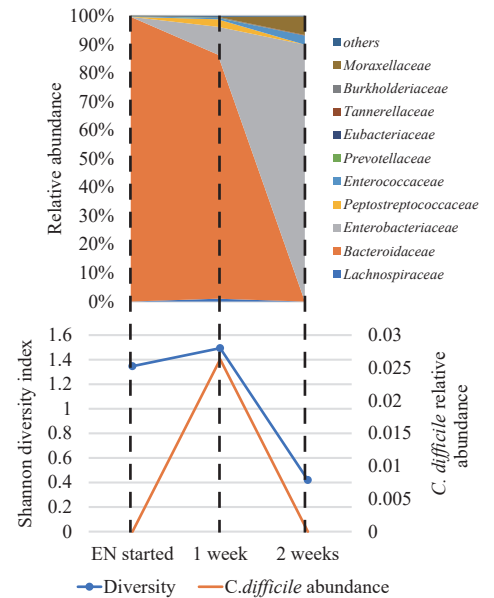

Patient 108 (CDC→CDI)

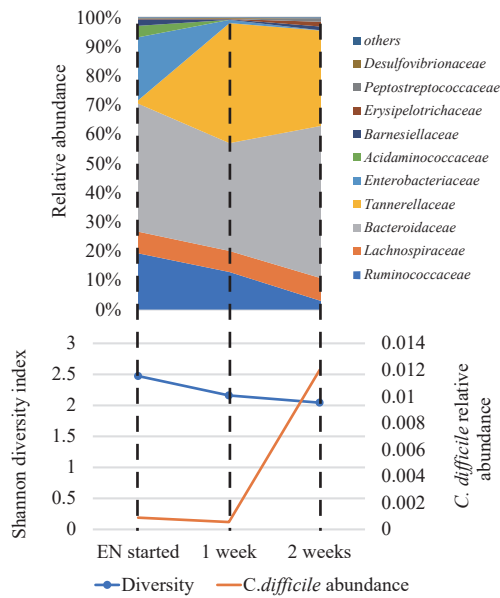

Patient 123 (CDI)

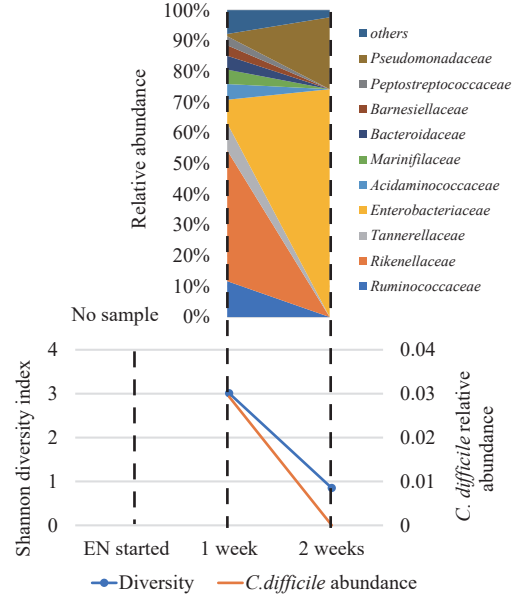

Patient 127 (CDI)

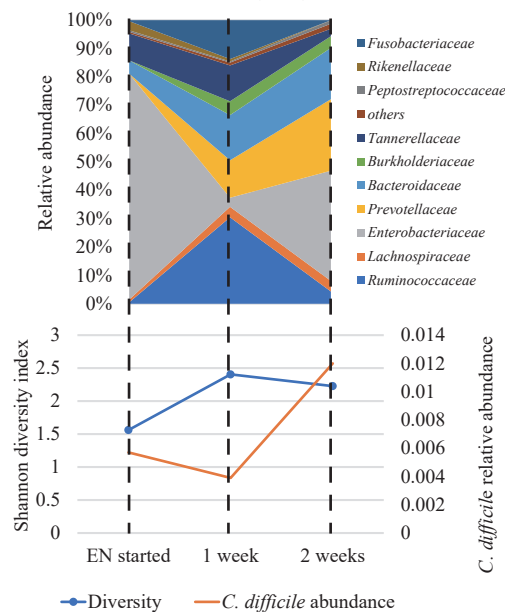

Patient 134 (CDI)

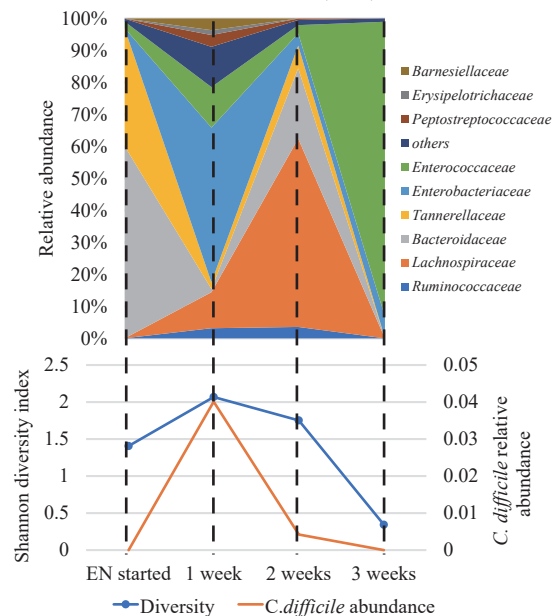

Supplement: Supplementary file 5 — Additional file 5 : Figure S2. Intestinal microbiota dynamics in C. difficile positive (CDP) patients. For each panel, changes in microbial composition at the family level are illustrated on the above axis, corresponding to the alterations in the C. difficile load (right) and microbial diversity (left) on the same timeline shown below. [file 13054_2020_3119_MOESM5_ESM.pdf]

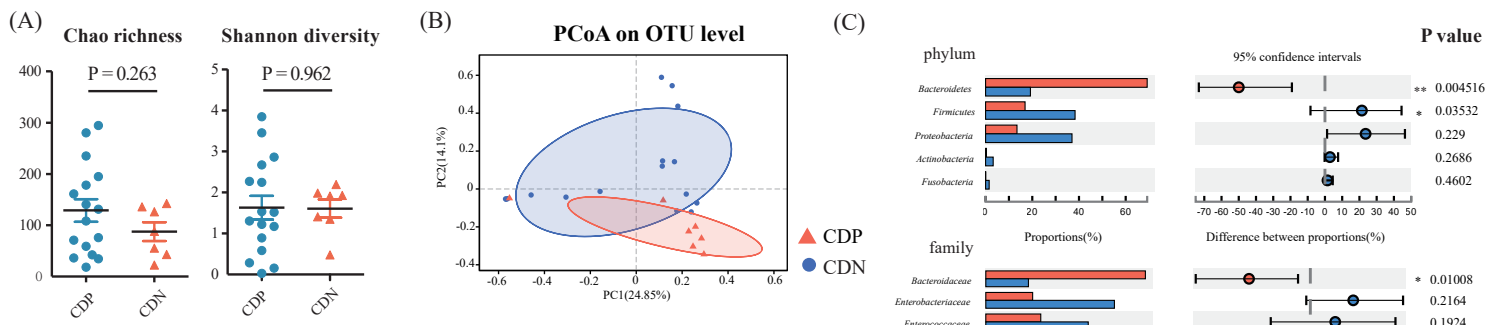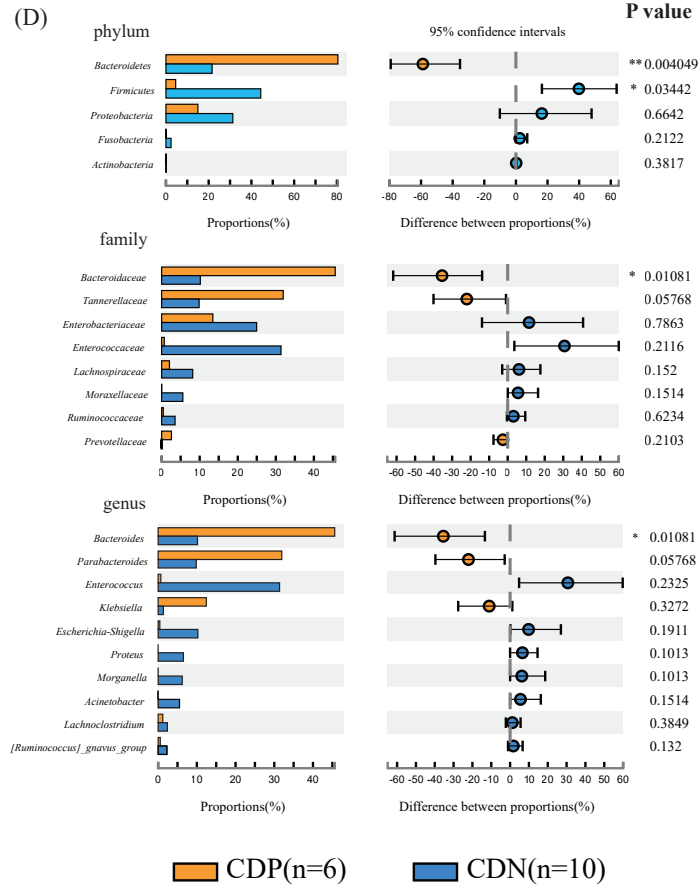

(E)

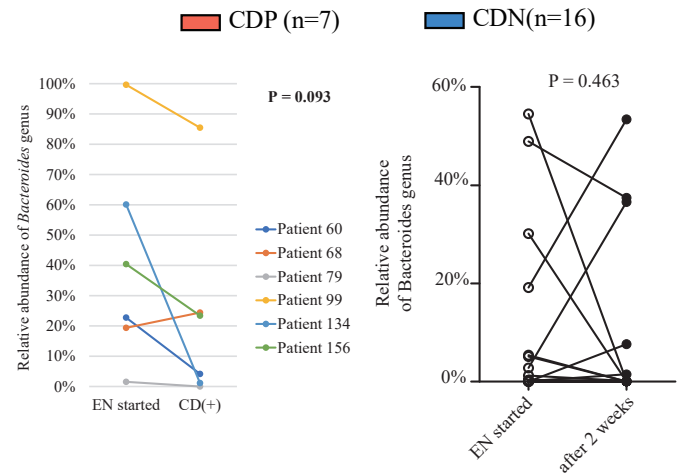

(F)

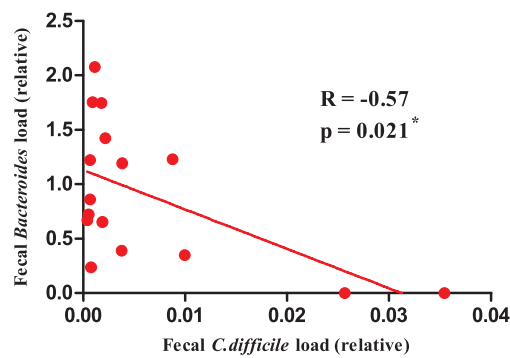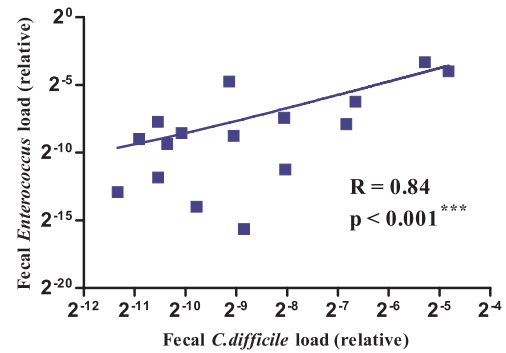

Supplement: Supplementary file 6 — Additional file 6 : Figure S3. Role of Bacteroides in CDI (A) and (B) The comparation in the composition of intestinal microbiota between CDP (n = 7) and CDN (n = 16) patients at the beginning of EN. (A) Differences of microbial richness and diversity, tested using Student’s t-tests. (B) Principal coordinates analysis plots based on the Bray-Curtis distance. (C) and (D) Wilcoxon rank sum tests were performed to analyze the differences between 7 CDP and 16 CDN patients (C) or those excluded because they were treated with metronidazole within 3 days of onset of EN (D). (E) Relative abundance of Bacteroides genus from the onset of EN to the first presence of C. difficile for CDP patients (n = 6; left), or from the onset of EN to 2 weeks later for CDN patients (n = 16; right). Differences were evaluated by Wilcoxon signed-rank test. (F) Correlation between the relative abundance of Bacteroides or Enterococcus and C. difficile calculated using qPCR, and analyzed using a Spearman’s correlation test. *P < 0.05, **P < 0.01. [file 13054_2020_3119_MOESM6_ESM.pdf]
